# Coiled-coil formation of the membrane-fusion K/E peptides viewed by electron paramagnetic resonance

**DOI:** 10.1371/journal.pone.0191197

**Published:** 2018-01-19

**Authors:** Pravin Kumar, Martin van Son, Tingting Zheng, Dayenne Valdink, Jan Raap, Alexander Kros, Martina Huber

**Affiliations:** 1 Department of Physics, Huygens-Kamerlingh Onnes Laboratory, Leiden University, Leiden, The Netherlands; 2 Department of Supramolecular and Biomaterials Chemistry, Leiden Institute of Chemistry, Leiden University, Leiden, The Netherlands; University of Dundee, UNITED KINGDOM

## Abstract

The interaction of the complementary K (*Ac-(KIAALKE)*_*3*_*-GW-NH*_*2*_) and E (*Ac-(EIAALEK)*_*3*_*-GY-NH*_*2*_) peptides, components of the zipper of an artificial membrane fusion system (*Robson Marsden H*. *et al*. *Angew Chemie Int Ed*. *2009*) is investigated by electron paramagnetic resonance (EPR). By frozen solution continuous-wave EPR and double electron-electron resonance (DEER), the distance between spin labels attached to the K- and to the E-peptide is measured. Three constructs of spin-labelled K- and E-peptides are used in five combinations for low temperature investigations. The K/E heterodimers are found to be parallel, in agreement with previous studies. Also, K homodimers in parallel orientation were observed, a finding that was not reported before. Comparison to room-temperature, solution EPR shows that the latter method is less specific to detect this peptide-peptide interaction. Combining frozen solution cw-EPR for short distances (1.8 nm to 2.0 nm) and DEER for longer distances thus proves versatile to detect the zipper interaction in membrane fusion. As the methodology can be applied to membrane samples, the approach presented suggests itself for in-situ studies of the complete membrane fusion process, opening up new avenues for the study of membrane fusion.

## Introduction

All living organisms utilize membrane fusion for their normal functioning. Cellular activities that involve membrane fusion are hormone secretion, enzyme release, neurotransmission etc. Membrane fusion needs a specialized set of proteins, such as the SNARE protein complex [1–7] (SNARE: soluble NSF attachment protein receptor; NSF = *N*-ethylmaleimide-sensitive factor). Membrane fusion induced by SNARE involves the coiled-coil interaction between three complementary SNARE proteins [8,9].

**Fig 1:**
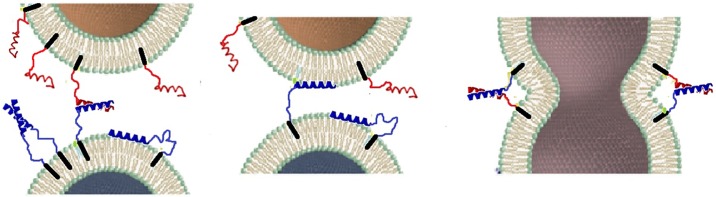
Membrane fusion model: Two membranes (top and bottom) are brought together by the lipidated peptide constructs (K/E): lipid anchor, black; PEG12 chains, the linker (for clarity drawn at the same color as the peptide); complementary peptides K, blue, and E, red, respectively. The proposed interaction [by Rabe *et al*., [14]] of peptide K with lipid head groups is also shown.

To understand protein-mediated membrane fusion, a coiled-coil model system mimicking the complex of SNARE proteins was designed [10–13]. It performs fusion by a pair of complementary lipidated oligopeptides K/E, which contain a lipid-anchor segment, a coiled-coil-zipper segment, and a linker that connects the two segments (see Fig 1).

To gain a better picture on membrane fusion, we focus on the coiled-coil zipper segment of the complex, which consists of the helical peptides K and E, for sequences see Table 1.

**Table 1:** Sequence of the zipper peptides E and K and EPR samples.

**peptides**	**sequences**
**E**	Ac-(EIAALEK)_3_-GY-NH_2_
**K**	Ac-(KIAALKE)_3_-GW-NH_2_
**E-SL**	Ac-(EIAALEK)_3_-GYC(SL)-NH_2_
**K-SL**	Ac-(KIAALKE)_3_-GWC(SL)-NH_2_
**SL-K**	Ac-C(SL)-(KIAALKE)_3_-GW-NH_2_
**samples used for EPR measurements**	
**E-SL**	
**K-SL**	
**SL-K**	
**E-SL:K-SL**	
**E-SL:SL-K**	

C(SL): cysteine with MTSL attached, Ac: acetyl

**Fig 2:**
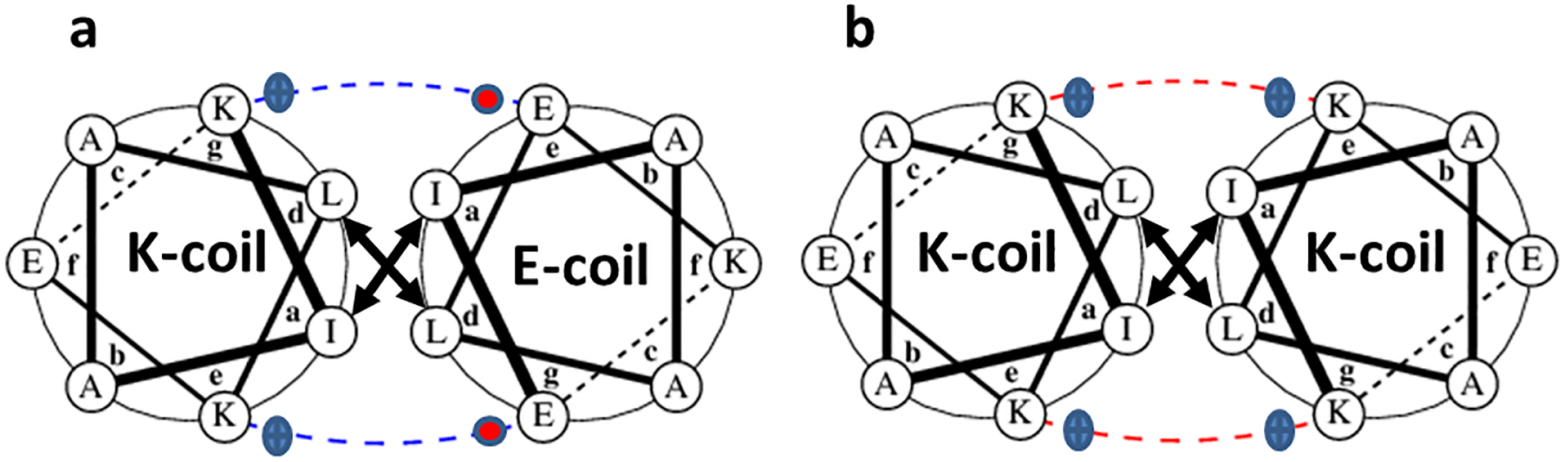
Helical wheel representation of the quaternary structure of parallelly oriented (a) K/E-heterodimer and (b) K-homodimer. **a**, **b**, **c**, **d**, **e**, **f**, and **g** indicates the position of heptad repeats. Blue dashed line: coulomb attractions of the positively charged lysine side chains and negatively charged glutamate side chains; Red dashed line: coulomb repulsions of the positively charged lysine side chains; bold arrows: Van der Waals interactions of hydrophobic leucine and isoleucine side chains. Helical wheel projections also showing the knobs into hole model [18]: Ile represents the hole; Leu represents the knob.

The peptides are relevant to initiate the interaction of the two membranes to be brought together, a process that relies on wrapping of the two α-helical amphipathic peptides around each other in a left-handed twist manner. To direct this process the coiled-coil peptides contain a characteristic seven residue repeat (**a**.**b**.**c**.**d**.**e**.**f**.**g**)_n_, where hydrophobic residues reside at positions **a** and **d** and polar residues at other positions as shown in Fig 2. The pattern of hydrophobic and polar residues in the amino acid sequence and the geometric properties of buried amino acid side chains influence the overall structure of these coiled-coils [15]. These structures are stabilized by an extensive network of hydrophobic interactions by the knobs-into-hole principle and by electrostatic interactions between cationic and anionic residues across the dimerization interface (see Fig 2).

Previous studies [16,17] have shown that the K- and E-peptides behave differently in solution: E, anionic in nature, is in a random coil conformation and K, cationic in nature, has some helical character and a larger tendency to self-aggregate than E.

A new mechanism was proposed about a twofold role of the K-peptide in membrane fusion [14,19]: a. to first bring the target vesicles into close proximity (≈ 8 nm) by K/E coiled-coil formation and b. to interact with the two opposing membranes resulting in lipid reorganization and promoting protrusion of the lipid acyl chains as the initial state of lipid mixing. In addition, the role of K-peptide induced membrane dehydration is not yet clear.

Circular-Dichroism (CD) experiments revealed the folding, i.e., formation of interacting α-helices of the two peptides in aqueous solution [17,20,21], showing that the wild-type proteins assemble into K/E heterodimeric coiled-coils with a folding constant of 1.8 x 10^7^ M^-1^ [20,22]. Some tendency for self-association was also found: For the pure E-peptides, a folding constant of 5.3 x 10^2^ M^-1^[20,22]and for the pure K-peptides, a folding constant of 3.4 x 10^3^ M^-1^ [20] was determined. The folding constant for the K-peptide suggests that it forms a homodimer under the conditions of the present study.

For the K/E heterodimer, techniques like Förster resonance energy transfer (FRET) and paramagnetic proton nuclear magnetic resonance (NMR) were applied to study the orientation of the heterodimer suggesting a parallel arrangement of the K and E helix [21]. Homodimers of K, predicted by the CD experiments were not yet characterized by these techniques.

Here, we investigate variants of the oligopeptides E and K, synthesized [21,23] following the original protocol developed by Litowski and Hodges [17] with spin labels attached (Table 1) to perform EPR experiments. We use continuous-wave (cw) EPR and the established pulsed EPR method called double electron-electron resonance (DEER) [24] at low temperatures to study the structure and the orientation of dimers of the E- and the K-peptides. Continuous-wave (cw) EPR and pulsed EPR have become important tools to investigate structural aspects of bio-macromolecular complexes. Double electron-electron resonance (DEER) is widely used in bio-chemical research and has been used to characterize biomolecular assemblies and to detect conformational changes in biomolecules as reviewed in [25,26]. Also, several reports of DEER on coiled-coil interactions appeared [27–30].

The EPR methodology applied on frozen solutions monitors the interpeptide dipolar interaction between the spin labels, and thereby the proximity of the spin-labelled regions of the peptides K and E. To investigate whether these peptide interactions can be detected by room temperature cw-EPR, we also performed these experiments. Room temperature cw-EPR uses the rotation-correlation time, which, since it depends on the size of the object studied, will increase as the peptides form dimers. These experiments are described in S1 Text.

In the present study, we demonstrate that, similar to the results of other techniques [21,31], the heterodimer of K/E peptides has a parallel orientation. We also report direct evidence for homodimers of the K-peptides and determine that the peptides in these homodimers adopt a parallel orientation.

## Materials and methods

### Peptide synthesis, labelling, and sample preparation

The synthesis of the MTSL-labelled K- and E-peptides listed in Table 1 has been described elsewhere [21,23]. Solutions of each peptide were prepared in phosphate buffered saline (PBS) buffer containing 20% (wt) glycerol used as a cryo-protectant for the preparation of frozen samples listed in Table 1. For studying the K/E coiled-coil-complex formation the two different peptides were mixed in equimolar amounts while keeping the total peptide concentration constant at 0.3 mM. Peptide solutions were put into 3 mm (outer diameter) quartz tubes and the samples were plunged into liquid nitrogen for fast freezing. The same samples were used for cw-EPR and DEER measurements. The degree of spin-labelling was determined by comparing the peptide concentration to the double integral of the EPR spectrum of the peptide calibrated with a reference sample of a spin label with known concentration. For E-SL and SL-K the spin-labelling degree was around 80%, for K-SL around 70%. Note that double integration introduces errors in the order of 15% of these values.

### cw-EPR measurements at 120 K

The cw-EPR measurements were performed at 9.7 GHz using an ELEXYS E680 spectrometer (Bruker, Rheinstetten, Germany) with a rectangular cavity (ER 4102 ST), using a modulation frequency of 100 kHz. For measurements at 120 K, a helium-gas flow cryostat (Oxford Instruments, United Kingdom) with an ITC502 temperature controller (Oxford Instruments, United Kingdom) was used. The frozen samples were inserted in the pre-cooled helium-gas flow cryostat. The EPR spectra were recorded using modulation amplitude of 0.25 mT and a microwave power of 0.63 mW. Typical accumulation times were 10–14 min. No absolute calibration of g values was made. For the simulation a constant shift was applied to the B_0_ field to account for the difference between measured B_0_ values and the actual B_0_ values at the sample.

### Simulation of EPR spectra

The spectral simulation was performed using Matlab (7.11.0.584, Natick, Massachusetts, U.S.A) and the EasySpin package [32]. For all simulations, the following spectral parameters were used: g = [2.00906, 2.00687, 2.00300] [27], the hyperfine tensor parameters A_XX_ = A_YY_ = 13 MHz, and the A_ZZ_ parameter was varied. We used A_ZZ_ = 103 MHz for **E-SL** and 102 MHz for both **K-SL** and **E-SL: K-SL**. The linewidth parameter (lwpp, peak to peak linewidth in mT) was obtained from the simulation of the spectrum of a sample of MTSL in the buffer described above and was kept fixed for the simulation of the other spectra. A traceless-dipolar tensor of the form [- D—D + 2D], in which 2D represents the parallel component of the dipolar tensor, was used. The value of D (in MHz) was varied until the simulation agrees with the experimental spectrum. By doing this we were able to obtain the dipolar frequency (in MHz), from which the corresponding inter-spin distance is calculated.

### DEER measurements

All DEER experiments were done at 9.7 GHz on an ELEXSYS E680 spectrometer (Bruker, Rheinstetten, Germany) using a 3 mm split-ring resonator (ER 4118XMS-3-W1). We performed the measurements at 40 K with a helium gas flow using a CF935 cryostat (Oxford Instruments, United Kingdom). The pump and observer frequencies were separated by 70 MHz and adjusted as reported before [33]. The power of the pump-pulse was adjusted to invert the echo maximally [24,34–36]. The length of the pump-pulse was set to 16 ns. The pulse lengths of the observer channel were 16 and 32 ns for π/2- and π—pulses, respectively. A phase cycle (+ x)—(- x) was applied to the first observer pulse. The complete pulse sequence is given by: ${\frac{\pi }{2}}_{obs}-\;\tau_{1}-\pi_{obs}-t-\pi_{pump}-(\tau_{1}+\tau_{2}-t)-\pi_{obs}-\tau_{2}-echo.$ The DEER time traces for ten different τ_1_ values spaced by 8 ns starting at τ_1_ = 200 ns were added to suppress proton modulations. Typical accumulation times per sample were 16–20 hours.

### DEER analysis

In order to analyze the DEER traces and extract the distance distributions, the software package “DeerAnalysis 2011” was used [37]. All the DEER traces were corrected by a homogeneous 3D-background function, which describes the three-dimensional random distribution of nano-objects in the sample [24,35,37]. This approach is justified because the proteins are soluble in buffer and no membranes are present. Peptides or proteins interacting with membranes can cause lower dimensionality background functions, such as a 2D background. The distance distribution was derived by the model-free Tikhonov regularization [24,34–37].

### Modeling of the dimer structure

In order to visualize the dimer of E-SL: SL-K, a model of the dimer was created using VMD software (Molecular Simulations). VMD software is owned by the Theoretical and Computational Biophysics Group, NIH Center for Macromolecular Modeling and Bioinformatics, at the Beckman Institute, University of Illinois at Urbana-Champaign.

## Results

**Fig 3:**
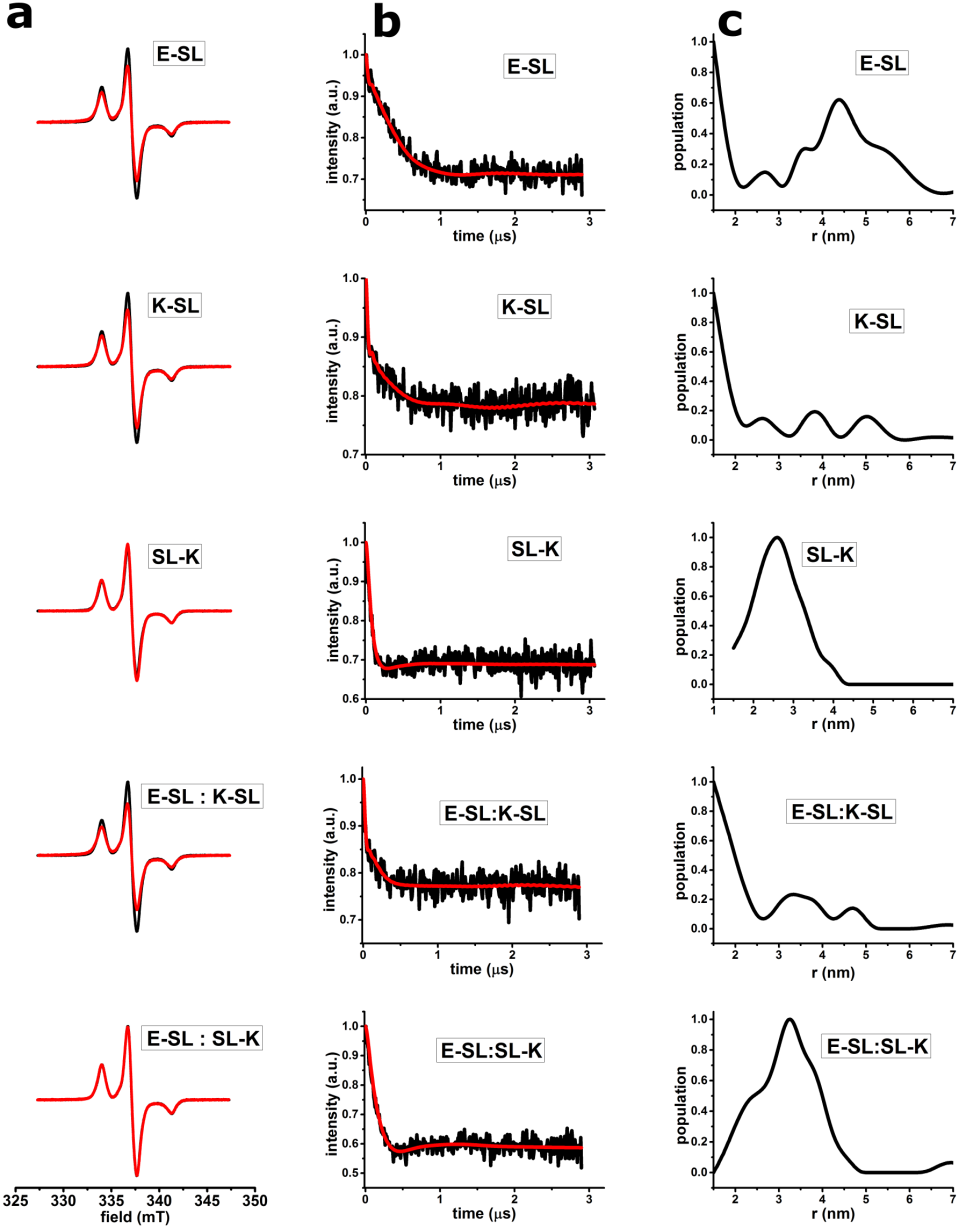
cw-EPR and DEER results of spin-labelled E- and K-peptides. (a) cw-EPR spectra of spin labelled E, K and coiled-coil K/E peptides at 120 K. Superposition of spectra of **E-SL**, **K-SL, SL-K, E-SL: K-SL,** and **E-SL: SL-K** peptides (red) is shown with the spectrum of pure MTSL (black). All spectra are normalized to the same number of spins. (b) DEER time traces of spin labelled E- and K-peptides (listed in Table 1) after background correction (black line), fit of the time trace (red line) with the distance distributions shown in c; c. distance distributions obtained after Tikhonov regularization. For raw DEER time traces, see S1 Fig.

Fig 3 shows the results of the frozen solution EPR experiments for the samples listed in Table 1. In Fig 3a, the cw-EPR spectra, and in Fig 3b and 3c the DEER results are displayed. The cw-EPR spectra (Fig 3a) of the samples are shown in red and are superimposed on the cw-EPR spectrum of MTSL measured under the same conditions as a monomeric reference. Fig 3a shows that the spectra of **E-SL**, **K-SL,** and of **E-SL**: **K-SL** are broadened with respect to MTSL, whereas spectra of **SL-K** and **E-SL: SL-K** are not.. These observations are summarized in Table 2.

**Table 2:** Summary of EPR properties and distances of K/E peptides.

samples	cw-EPR line- broadening compared to MTSL	distance from cw-EPR spectra (nm)	modulation depth of the DEER trace	distance from DEER analysis (nm) (width (FWHM))
**E-SL**	yes	1.8–2.0	0.29	4.4 (1.6)
**K-SL**	yes	1.8–2.0	0.21	na
**SL-K**	no	no short distances	0.31	2.6 (1.4)
**E-SL**: **K-SL**	yes	1.8–2.0	0.22	na
**E-SL**: **SL-K**	no	no short distances	0.40	3.2 (1.3)

na: modulation depth too low to obtain relevant distances (see text); FWHM: full width half maximum of distance peak

In Fig 3b the DEER time traces are shown after background correction together with the fits corresponding to the distance distributions in Fig 3c. The raw DEER data are shown in S1 Fig.

The DEER traces of **K-SL** and the 1: 1 mixture of **E-SL**: **K-SL** (shown in Fig 3b) have a low modulation depth (see Table 2), showing that a significant population of the peptides has spin pairs with distances outside the DEER measurement range, i.e., smaller than 2 nm or larger than 5 nm. The sharp feature in the first 0.05 ns of the DEER curve has been interpreted before as a contribution of short distances [38]. That such short distances are indeed present is evidenced by the cw-EPR results. The origin and the consequences of low modulation depth are analyzed in the Discussion section.

In contrast, the modulation depth in the DEER traces of **E-SL**, **SL-K** and **E-SL**: **SL-K** (see Table 2) shows that a significant population of spin pairs in these samples have distances in the sensitive range of DEER: The distance distributions of **SL-K**, and **E-SL: SL-K** (for parameters, see Table 2) show well defined peaks, the width of which, 1.4 nm and 1.3 nm (full width half maximum (FWHM), suggests that multiple conformations contribute to the distance distribution [39]. For **E-SL**, the width of the peak is even larger (1.6 nm FWHM, see Table 2). For **SL-K**, the smaller width of the distance peak could also in part be caused by the lower sensitivity of DEER to distances below 2 nm, as described for example in [40].

Combining the results of cw-EPR and DEER in Table 2 the distances obtained can be summarized as follows: In the K**-SL** and **E-SL**: **K-SL** samples only distances in the range of 1.8–2.0 nm are observed. These short distances manifest themselves in the line broadening in cw-EPR. Distances longer than 2.0 nm do not occur in these samples as evidenced by the low DEER modulation depth. The **SL-K**, and **E-SL: SL-K** samples show the opposite behavior. They have no short distances, i.e. distances between 1.8 and 2.0 nm, however they have significant DEER modulation depth. The results of the DEER measurements are a distance of 2.6 nm for **SL-K,** and 3.2 nm for **E-SL**: **SL-K.** The **E-SL** peptide has a distance of 4.4 nm (obtained from DEER measurements) and a short distance in the range of 1.8 to 2.0 nm (obtained from cw-EPR) suggesting a broad range of distances, as we will discuss in detail below. Room temperature cw-EPR was performed to test, if under those conditions dimer formation can be detected. Overall the effects were weak, and are described in S1 Text.

## Discussion

In this study, we determine the interactions between the K- and the E-peptides by EPR using two complementary methods: Short distances (up to 2 nm) are detected by cw-EPR and longer distances by DEER. For these experiments, the peptides are investigated in frozen solution using the same sample for both types of measurements. Table 2 summarizes the results obtained.

The broadening of the cw-EPR spectra for **E-SL**, **K-SL** and **E-SL**: **K-SL** corresponds to distances in the range of 1.8 to 2.0 nm, as derived from simulations of the EPR spectra under conditions described in Materials and methods.

In the present samples, because of different spin-label linker conformations and the intermolecular nature of the interactions, cw-EPR line broadening most likely reflects not a single distance/conformation and therefore only a range of distances (see Table 2) can be derived from the broadening observed. Longer distances (> 2 nm) are detected by DEER, a method that gives the distance distributions, which reflect the distances of all members of the ensemble. Under the present experimental conditions, distances longer than 5 nm cannot be reliably detected. In DEER, the modulation depth reflects the fraction of the sample in which two spins interact within the distance range of the experiment, here 2–5 nm. In the present systems, with intermolecular interactions and multiple conformations, the distances found in the distance distributions of DEER experiments with low modulation depth, i.e., below 0.25, see Table 2, are not meaningful, because these distances are not representative of a major fraction of the spins in the sample. In DEER experiments on isolated proteins and on specific protein-protein complexes, the quantitative analysis of the results can be extended significantly. In the present case, however, the non-covalent interaction of the peptides probed and the differences in labelling degree of the peptides (Materials and methods) would make such a detailed analysis questionable.

**Fig 4:**
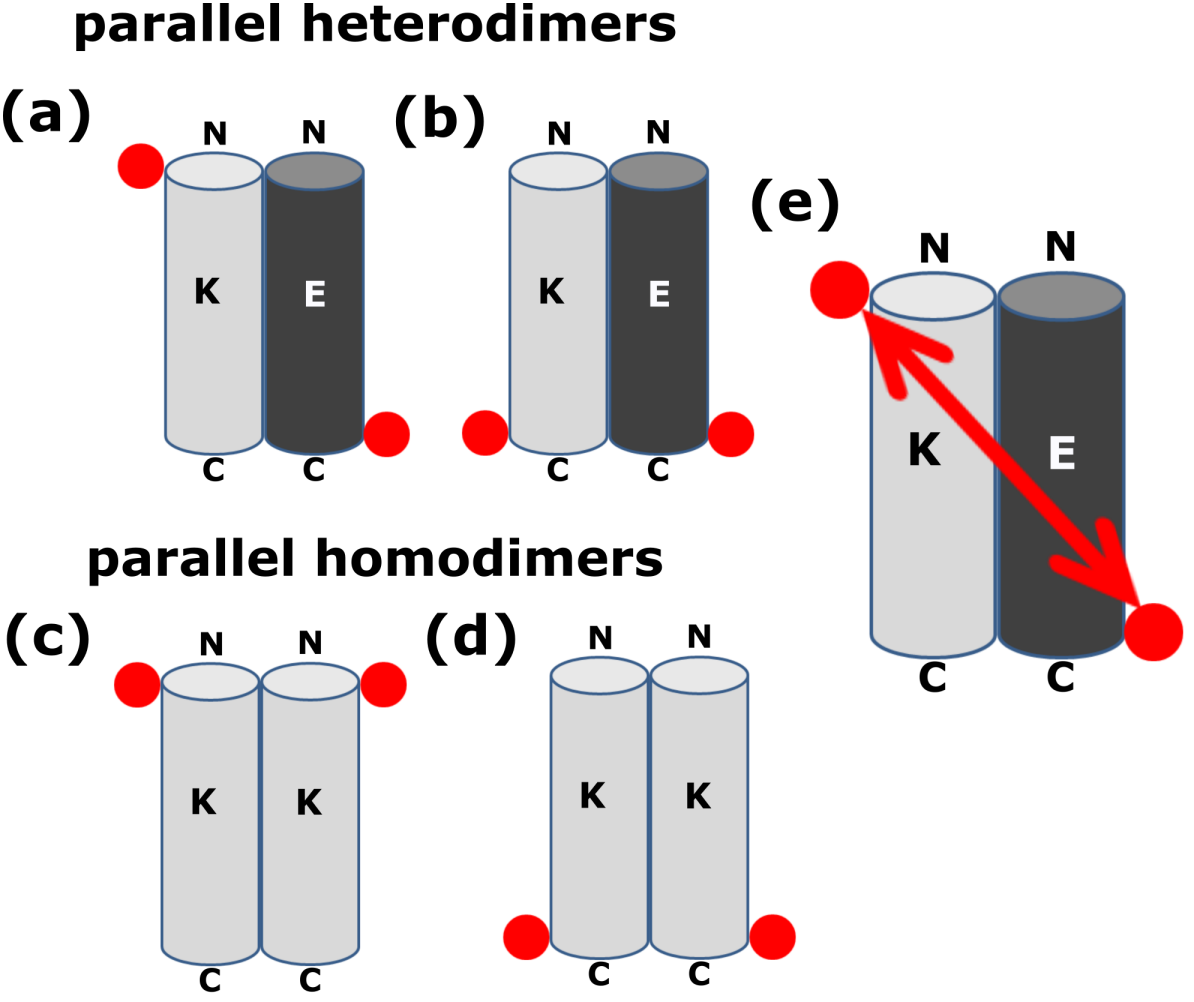
Schematic representation of the quaternary structure of the K/E heterodimer and K homodimer, both in parallel arrangement. K- and E-peptides are shown as cylinders in grey and black color respectively. Red circle represents the spin-label position. (a) K/E heterodimer, with the N-terminus of K- and the C-terminus of E-peptide spin labelled, while in case of (b), the C-terminus of both peptides are spin labelled. (c) and (d): parallel K homodimers. (c) K-peptide labelled at the N-terminus, (d) at the C-terminus. (e) shows schematically the distance between two spin labels in case of the parallel K/E-heterodimer where the N-terminus of K- and the C-terminus of E-peptide is spin labelled (situation (a)).

To facilitate the following discussion, Fig 4 schematically introduces the distances observed for N- or C-terminally labelled peptides for parallel homo- and heterodimers.

To briefly summarize: Heterodimers, in which one partner is labelled at the N- and other at the C-terminus give a **long** distance (Fig 4(a) and 4(e)). Heterodimers, in which both partners are labelled at the C-terminus give a **short** distance (Fig 4(b)), the same result holds for the case in which both partners are labelled at the N-terminus. The two experimentally realizable situations for parallel homodimers are: labelling at the N-terminus (Fig 4(c)) or labelling at the C-terminus (Fig 4(d)), and both result in **short** distances. Using these principles we arrive at the following interpretations:

### K/E heterodimers

**Fig 5:**
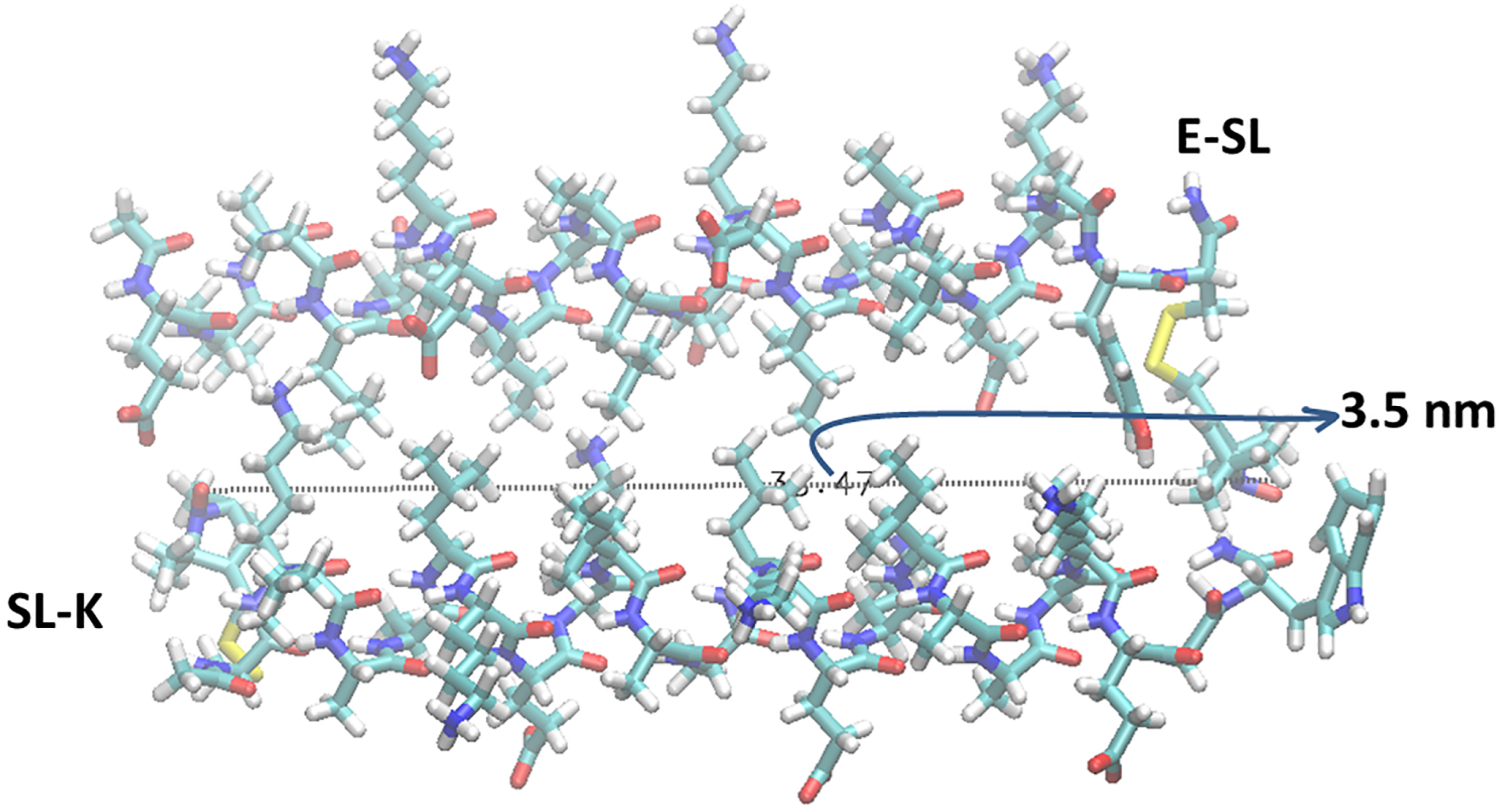
Quaternary structure of the K/E heterodimer based on VMD software (Molecular Simulations). Top: E-SL; Bottom: SL-K. Relative orientation: parallel with Ile and Leu oriented towards the center of the K/E complex. The arrow indicates the distance between the spin labels attached to E- and K-peptide in the dimer form.

The K/E mixture shows a short distance detected in cw-EPR, i.e. between 1.8 and 2.0 nm, when the C-termini of both peptides are labelled (**E-SL: K-SL**). A distance of 3.2 nm is observed, when the C-terminus of E and the N-terminus of K are labelled (**E-SL: SL-K**) (see Results section). A short distance between the two C-terminally labelled peptides (**E-SL: K-SL**) and a longer one, when one peptide is labelled at the C-terminus and the other at the N-terminus (**E-SL: SL-K**) is in qualitative agreement with a parallel heterodimer situation as shown in Fig 4(b), 4(a) and 4(e), respectively. The relatively large width of the DEER distance distribution of **E-SL: SL-K** suggests more than a single conformation of the dimer. In order to visualize the dimer of **E-SL**: **SL-K**, a model of the dimer was created with the VMD software (Molecular Simulations) assuming standard φ and ψ angles for a regular α helix including cysteine (SH) (both Cys-K and E-Cys). We have built the S-S bond and included the atoms required to define the MTSL label. The molecules were arranged as close as possible and in a parallel orientation (shown in Fig 5) to represent the quaternary structure of the K/E dimer.

The dimer of **E-SL**: **SL-K** with spin labels attached gives a distance of 3.5 nm between the nitroxide groups of the spin label in agreement with the results of the DEER experiments (Table 2). The quaternary structure of the parallel heterodimer was also confirmed previously by different techniques (H-NMR, PRE-NMR, FRET) [21,31], which reveal not a single conformation but an ensemble of conformations for this dimer. The model shown in Fig 5 is just one member of this ensemble. Considering these findings, our present result is in full agreement with a parallel heterodimer model.

#### K-peptide homodimers

The K-peptide sample shows a short distance, detected by cw-EPR, when labelled at the C-terminus (K-SL) and a distance of 2.6 nm when labelled at the N-terminus (SL-K). A distance of 1.8–2.0 nm for K-SL is only compatible with a parallel homodimer. The distance for SL-K, 2.6 nm, is consistent with a parallel homodimer in which the spin labels point away from each other and possibly indicate flared-out ends of the N-terminal region of the homodimer. The finding of a K homodimer based on EPR, in our study, was not described before. We attribute the longer distance between the spin labels at the N-terminus (SL-K) to fraying of the helix ends in combination with the spin labels pointing away from each other. On the other hand, the short distance at the C-terminus (K-SL), which contains a tryptophan residue, can be explained by the H-bond formation between the NO of the spin label with the NH proton of the indole group of the tryptophan. This interaction might stabilize the C-terminus and avoid fraying at this end of the peptide [41,42].

The finding of a parallel homodimer for the K-peptide must be attributed to dominance of the knob-into-hole interaction (as depicted in Fig 2b), which overrides the unfavorable electrostatic interaction of the K-residues and the repulsive helix-dipole-dipole interaction between the individual helices the parallel homodimer. Using the formalism to calculate the helix-dipole interaction described in [43], we obtain dipole interaction energies of 53 kcal/mol for a parallel arrangement of two helices with a distance between the two long helix axes of 1.1 nm. As described in [44] the knob-into-hole interaction of the I (Ile) and L (Leu) residues at helix positions **a** and **d** must be sufficient to override this helix repulsion.

#### E-peptide interactions

The E-peptide, in contrast to the K-peptide, is not α-helical in solution. Circular dichroism (CD) studies [20,22] show the low tendency of the E-peptide to form dimers that is evident from its folding constant of 5.3 x 10^2^ M^-1^ [20,22]. Under the present experimental conditions, this would correspond to a population of 7% dimer. That means the E-peptide should not have defined spin-spin interactions in more than 7% of the population. The EPR properties of the E-peptide indeed differ from those of all the other samples in this study: The E-peptide shows pronounced short and long distances (see Table 2), which we interpret as a large range of conformations, or unspecific aggregation.

#### Room temperature EPR

The room temperature cw-EPR results are fully consistent with the frozen solution data; longer rotation-correlation times (τ_r_) are observed for K/E heterodimers, independent of which position is labelled (see S1 Text). This slower rotation derives from the increase in size when the heterodimer is formed. The presence of K homodimers explains why the increase in τ_r_ is less pronounced in K/E heterodimers in which K is spin labelled and E is added, compared to the samples where E is spin labelled: The K homodimers in the **K-SL** samples make the observed **K-SL** rotation-correlation time (τ_r_) longer than that of monomeric peptide, explaining why **K-SL** has a longer τ_r_ than **E-SL** (see Table A in S1 Text). As a result, the overall increase in τ_r_ is smaller when E-peptide is added to **K-SL** or **SL-K** than when K is added to **E-SL**. Local mobility of the spin label further dampens the effect of dimerization (see S1 Text).

## Conclusion

In conclusion, we show that the frozen solution EPR approach gives insight into the interaction of the K/E peptides, peptides that are designed for membrane fusion. As the methodology can be applied to membrane samples, the approach presented suggests itself for in-situ studies of the complete membrane fusion process, opening up new avenues for the study of membrane fusion.

## Supporting information

S1 Fig Raw DEER time traces of spin-labelled E- and K-peptides. Black line represents the raw DEER time traces before background correction, and red line the background.
(TIF)

S1 Text Continuous-wave EPR measurements at room temperature. (PDF)
