# Supplementary figures and images for: Coiled-coil formation of the membrane-fusion K/E peptides viewed by electron paramagnetic resonance
Source: PLoS One. 2018 Jan 19;13(1):e0191197. doi: 10.1371/journal.pone.0191197 (PMC5774749; doi:10.1371/journal.pone.0191197)

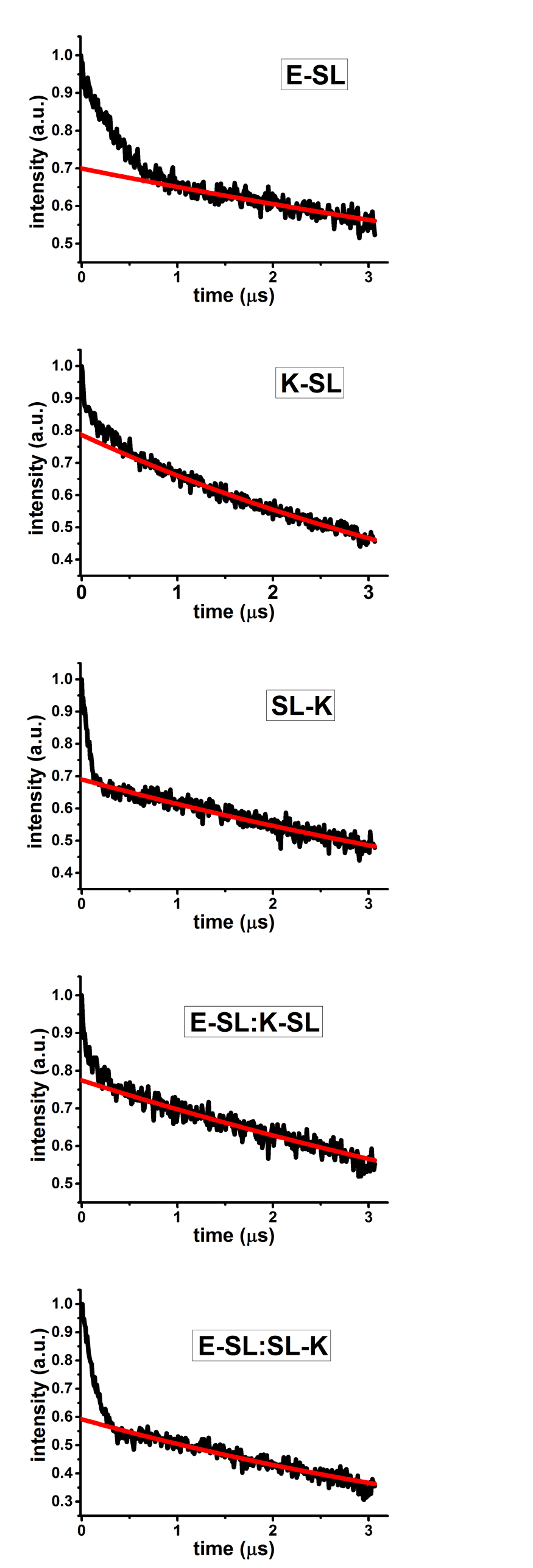

Supplement: S1 Fig — Black line represents the raw DEER time traces before background correction, and red line the background. (TIF) [file pone.0191197.s001.tif]
