# Supplementary material for: Coiled-coil formation of the membrane-fusion K/E peptides viewed by electron paramagnetic resonance
Source: PLoS One. 2018 Jan 19;13(1):e0191197. doi: 10.1371/journal.pone.0191197 (PMC5774749; doi:10.1371/journal.pone.0191197)
Supplement: S1 Text — (PDF) [file pone.0191197.s002.pdf]

S1 Text

Materials and methods of room temperature cw-EPR experiments

Samples were prepared in phosphate buffered saline (PBS), pH 7.4. Samples were made with different concentrations and combinations of K- and E-peptides, which we summarized in three categories: (i) samples with only labelled peptides with total peptide concentration of 150 μM, (ii) samples with mixtures of a labelled peptide with a non-labelled peptide, both 100 μM, and (iii) a mixture of a labelled peptide with another labelled peptide, both 100 μM. The cw-EPR measurements were performed with the same resonator as used in the measurements at 120 K (see main text). Measurements were performed at 20 ^{o}C, using 0.63 mW of microwave power, 100 kHz modulation frequency and a modulation amplitude of 0.04 mT. Total time to acquire EPR spectra was 20 min per sample.

Spectral simulation was performed using the EasySpin package [1]. For all simulations, the following spectral parameters were used: g = [2.0078 2.0058 2.0023], and the hyperfine tensor parameter A = [16.77 16.77 101.86] MHz. A second component was added (5%) to account for the satellite lines due to

coupling of the electron spin with ^{13}C (I = ½) nuclei in natural abundance. For this fraction A = [18.56 18.56 18.56] MHz was used. The linewidth parameters were kept fixed for the simulation within a group of particular labelled peptide (e.g. **E-SL**, **E-SL : E**, or **E-SL : K**). The rotation-correlation time (τ_r) was varied until the simulation agrees with the experimental spectrum.

Rotation-correlation time of the peptide

The rotation-correlation time (τ_r) of the peptide is calculated using the Stokes-Einstein equation

$$\tau_r = \frac{\eta V}{k_B T} \qquad (1)$$

where η is the viscosity of the solution, for water 1.00 mPa · s, k_B is the Boltzmann constant, and T is the temperature, which is 293 ± 1 K. The volume V of the E- and K-peptides is described by cylinders with a length of 3.9 nm and a diameter of 1.1 nm. The calculated volumes are 3.7 nm^3 for the monomeric peptide and 7.4 nm^3 for the dimer. Using the Stokes-Einstein equation, we calculated the τ_r values of 0.92 ns for a monomeric peptide and of 1.83 ns for a dimer.

Averaging of dipole-dipole interaction

For a system containing two unpaired electron spins, dipole-dipole interaction is averaged by molecular tumbling if

$$\omega_{dd} < \frac{2\pi}{\tau_r} \quad (2)$$

The dipole-dipole coupling (ω_{dd}) between two spins is proportional to the inverse cube of the distance [2]

$$\omega_{dd}(\theta, r) = \frac{2\pi g_1 g_2}{g_e^2}(3\cos^2\theta - 1)\frac{52.04}{r^3}[\text{MHz nm}^3] \quad (3)$$

where g_1 and g_2 are the g values of the two spins, g_e is the g value of the free electron, r is the distance between two spins, and θ is the angle between the spin-spin vector and the magnetic field. For τ_r = 1.83 ns an upper limit of ω_{dd} = $546 \cdot 10^6$ rad/s results, which corresponds to a distance of 0.8 nm.

Results and Discussion of room temperature cw-EPR experiments

Figure A shows the EPR spectrum of **E-SL : E** superimposed on the spectrum of **E-SL : K**. The spectra are superimposed such that the middle one of the three EPR lines overlaps optimally. The high-field line in the spectrum of **E-SL : K** is broadened compared to **E-SL : E**. A similar feature is observed in the spectra of

the samples in which **K-SL** or **SL-K** are mixed with their partner peptides (data not shown).

Simulations were performed with a model of isotropic rotation. The simulated spectra agree well with the experimental spectra, i.e., within the noise amplitude. The parameters obtained by the simulations are the rotation-correlation times (τ_r) and linewidths given in Table A.

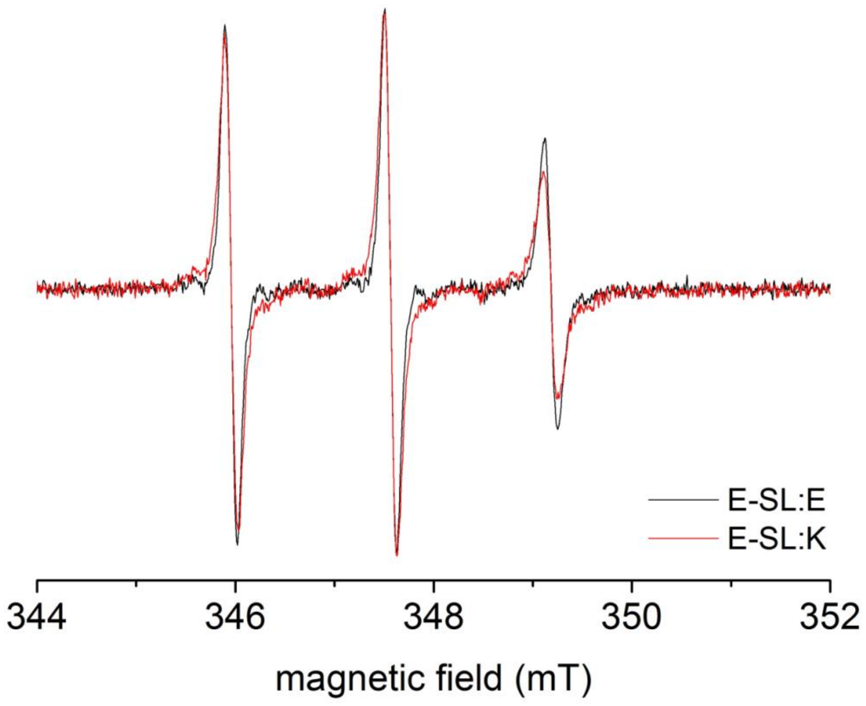

Figure A. The room temperature cw-EPR spectrum of **E-SL : E** (in black) superimposed on **E-SL : K** (in red).

Considering the three samples that contain **E-SL**, the τ_r values of **E-SL** and **E-SL : E** agree within the experimental error, whereas the τ_r of **E-SL : K** is significantly larger. The same is true for samples containing **K-SL** and **SL-K**. The

increase in τ_r is largest for **E-SL**, i.e., from 158 (**E-SL**) to 307 ps (**E-SL : K**), and smallest for **K-SL**. Amongst the heterodimers, τ_r is largest for **E-SL : K** (307 ps) and smallest for **K-SL : E** (236 ps).

Table A. Parameters used for the simulation of EPR spectra at room temperature

**sample**	**Linewidth[a] (mT)**	**τ_r[b] (ps)**
**E-SL**	0.11/ 0.02	158
**E-SL : E**	0.11/0.02	186
**E-SL : K**	0.11/0.02	307
**K-SL**	0.10/ 0.04	169
**K-SL : K**	0.10/ 0.04	181
**K-SL : E**	0.10/0.04	236
**SL-K**	0.09/ 0.07	197
**SL-K : K**	0.09/ 0.07	200
**SL-K : E**	0.09/ 0.07	269

[a] The spectral lines were best described using a mixture of Gaussian and Lorentzian lineshapes. The first and second value corresponds to the width of the Gaussian and Lorentzian line, respectively.

[b] The rotation-correlation time was determined with an error of ± 15 ps.

To check if the observed τ_r values relate mainly to the local mobility of the spin label, we calculate the rotation-correlation time of the peptide using the Stokes-Einstein equation (see Materials and methods of S1 Text). The measured τ_r values (Table A) are significantly smaller than those calculated for the rotation of the peptide itself, i.e., 0.92 ns for peptide K and 1.83 ns for the heterodimer, revealing that τ_r is largely determined by local mobility, i.e.,

rotation of the nitroxide about the single bonds joining it to the peptide and/or the mobility in the peptide backbone.

The τ_r changes reveal that the local mobility decreases when heterodimers are formed. The local-mobility change is largest for the C-terminus of the E-peptide, and also somewhat larger for the N-terminus of the K-peptide than for the C-terminus of K. The longer τ_r values observed for the K-peptides (**K-SL** and **SL-K**) compared to the E-peptide (**E-SL**) could be due to K-homodimer formation.

A significant increase of τ_r is found in all cases where heterodimers are formed, irrespective of the position of spin-label attachment (N- or C-terminus, E- or K-peptide), showing that mobility measurements by cw-EPR are sensitive to the formation in the K/E heterodimers.

To check, if spin-spin interaction can be observed in liquid solution, we measured mixtures of peptides in which both peptides are spin labelled. Figure B shows the overlay of the spectrum of **E-SL : K-SL** and the suitable reference spectrum, similarly Figure C for **E-SL : SL-K**. The spectra of samples in which both partners are labeled are identical within the noise to their respective reference spectra.

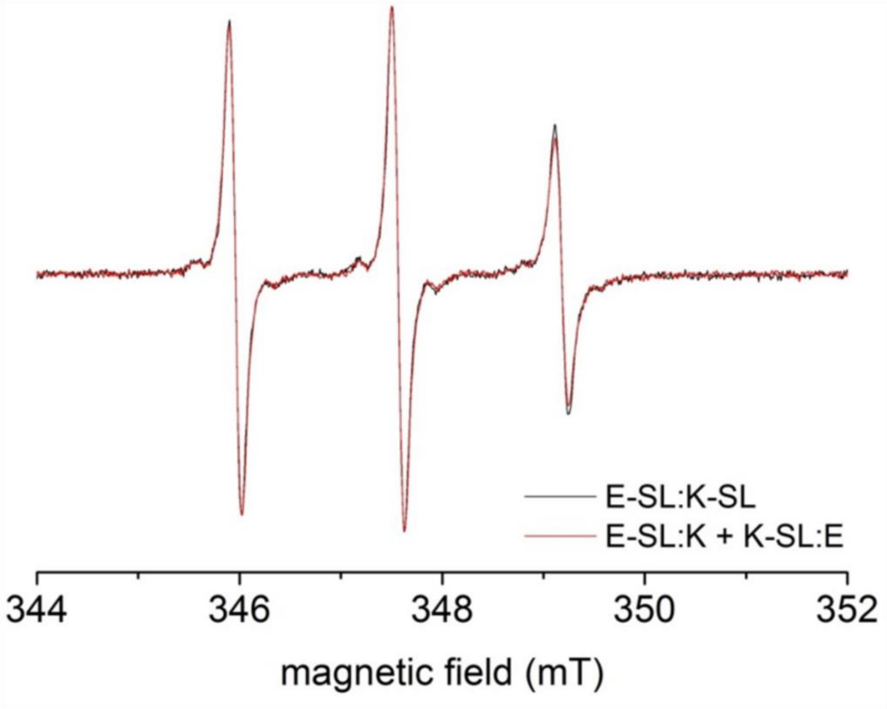

Figure B. The spectrum of **E-SL : K-SL** (in black) superimposed on the sum of the spectra of **E-SL : K** and **SL-K : E** (in red).

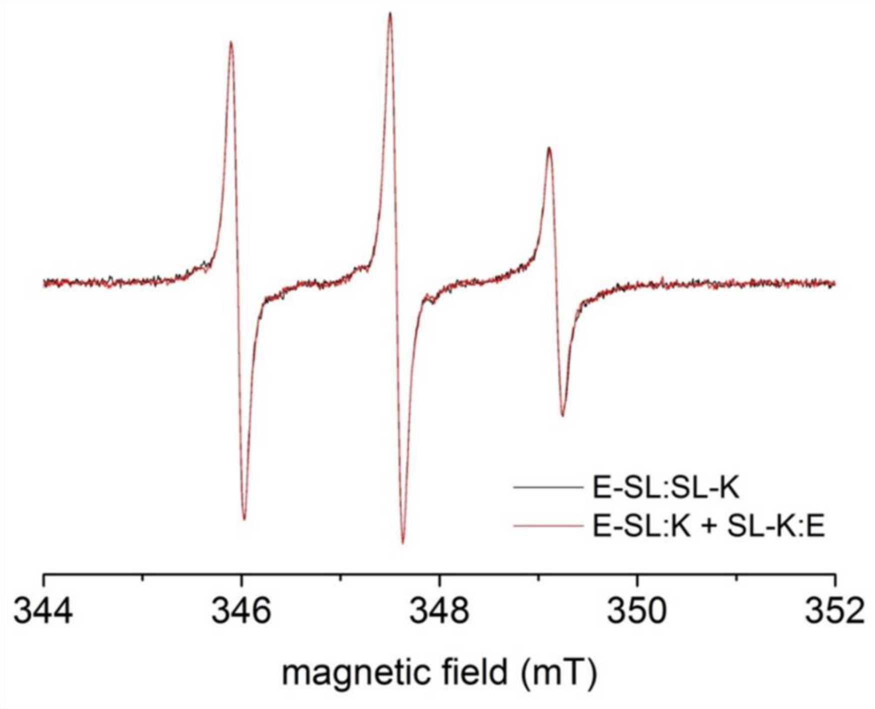

Figure C. The spectrum of **E-SL : SL-K** (in black) superimposed on the sum of the spectra of **E-SL : K** and **SL-K : E** (in red).

Thus, for none of the combinations of spin-labeled peptides (Figure B and C) spin-spin interactions are observed, showing that the spin labels are too far apart to have either exchange or dipolar interaction. Exchange interaction causes line broadening or extra lines in the EPR spectrum, if the distance between two spins is smaller than 0.5 nm. And, as it is an isotropic interaction, it should show up in the spectra. As no signature of exchange interaction is observed, the distance between the spins must be longer than 0.5 nm. The dipole-dipole interaction of the electron spins could be observed, if the dipolar interaction is sufficiently large not to be averaged by molecular tumbling. Using the τ_r expected for a peptide dimer, a dipolar interaction should be visible for distances smaller than 0.8 nm (see Materials and methods of S1 Text). The absence of any such effect on the spectra of the peptide partners, where both C-termini are labeled (**E-SL : K-SL**) or where the E-C-terminus and K-N-terminus (**E-SL : SL-K**) are labeled, shows that the spin labels are separated by more 0.8 nm. This result is fully consistent with the distances determined by the frozen solution measurements (see main text).

Finally, we conclude that frozen solution EPR in the combination of DEER and cw-EPR, as described in the main text, is more specific to determine dimerization of the peptides. In room temperature, liquid solution experiments,

the combination of local mobility of the spin label and averaging of the dipolar interaction reduce the effects of dimerization and make the outcome more ambiguous and difficult to interpret.

References:

1. Stoll S, Schweiger A. EasySpin, a comprehensive software package for spectral simulation and analysis in EPR. J Magn Reson. 2006;178:42–55.

2. Jeschke G. Distance measurements in the nanometer range by pulse EPR. ChemPhysChem. 2002;3:927–932.
